# Tumor Volume Estimation and Quasi-Continuous Administration for Most Effective Bevacizumab Therapy

**DOI:** 10.1371/journal.pone.0142190

**Published:** 2015-11-05

**Authors:** Johanna Sápi, Levente Kovács, Dániel András Drexler, Pál Kocsis, Dávid Gajári, Zoltán Sápi

**Affiliations:** 1 Research and Innovation Center of Obuda University, Physiological Controls Group, Obuda University, Budapest, Hungary; 2 Department of Control Engineering and Information Technology, Budapest University of Technology and Economics, Budapest, Hungary; 3 Preclinical Imaging Center, Gedeon Richter Plc., Budapest, Hungary; 4 1st Department of Pathology and Experimental Cancer Research, Semmelweis University, Budapest, Hungary; European Institute of Oncology, ITALY

## Abstract

**Background:**

Bevacizumab is an exogenous inhibitor which inhibits the biological activity of human VEGF. Several studies have investigated the effectiveness of bevacizumab therapy according to different cancer types but these days there is an intense debate on its utility. We have investigated different methods to find the best tumor volume estimation since it creates the possibility for precise and effective drug administration with a much lower dose than in the protocol.

**Materials and Methods:**

We have examined C38 mouse colon adenocarcinoma and HT-29 human colorectal adenocarcinoma. In both cases, three groups were compared in the experiments. The first group did not receive therapy, the second group received one 200 *μg* bevacizumab dose for a treatment period (protocol-based therapy), and the third group received 1.1 *μg* bevacizumab every day (quasi-continuous therapy). Tumor volume measurement was performed by digital caliper and small animal MRI. The mathematical relationship between MRI-measured tumor volume and mass was investigated to estimate accurate tumor volume using caliper-measured data. A two-dimensional mathematical model was applied for tumor volume evaluation, and tumor- and therapy-specific constants were calculated for the three different groups. The effectiveness of bevacizumab administration was examined by statistical analysis.

**Results:**

In the case of C38 adenocarcinoma, protocol-based treatment did not result in significantly smaller tumor volume compared to the no treatment group; however, there was a significant difference between untreated mice and mice who received quasi-continuous therapy (*p* = 0.002). In the case of HT-29 adenocarcinoma, the daily treatment with one-twelfth total dose resulted in significantly smaller tumors than the protocol-based treatment (*p* = 0.038). When the tumor has a symmetrical, solid closed shape (typically without treatment), volume can be evaluated accurately from caliper-measured data with the applied two-dimensional mathematical model.

**Conclusion:**

Our results provide a theoretical background for a much more effective bevacizumab treatment using optimized administration.

## Introduction

Bevacizumab (Avastin) [1] is an exogenous inhibitor, which inhibits the biological activity of human VEGF [2]. Several studies have investigated the effectiveness of bevacizumab therapy according to different types of cancer [3]: lung cancer [4, 5], breast cancer [6–8], colon cancer [9], renal cell carcinoma [10], gastric cancer [11], pancreatic cancer [12], prostate cancer [13] and melanoma [14]. The majority of debate over Avastin is about breast cancer because, in 2011, the US Food and Drug Administration (FDA) revoked the approval of Avastin for breast cancer treatment in the absence of decisive therapeutic benefit; however, several clinical trials suggested that Avastin can be effective in breast cancer treatment [6, 7].

The 1st Department of Pathology and Experimental Cancer Research of the Semmelweis University (Budapest, Hungary) and the Physiological Controls Group of the Obuda University (Budapest, Hungary) began collaborating on antiangiogenic therapy research in 2012—including the current experimental investigations. Small MRI measurements were done in the Preclinical Imaging Center of Gedeon Richter Plc. (Budapest, Hungary). The aim of the experiment was to create and validate a clinically relevant tumor growth model (using C38 colon adenocarcinoma), focusing on the effect of angiogenesis. Tumor growth was investigated without therapy and with antiangiogenic therapy (using bevacizumab [15]). Examination of tumor growth belongs not only to the basic medical research, but to the biomedical engineering field as well. Based on the experimental data, model identification can be carried out which describes the mathematical model of the investigated biological process. Using the mathematical model, different dosage algorithms can be designed for antiangiogenic cancer therapy [16–19]. Due to the collaboration between medical doctors and biomedical engineers, model-based treatment protocols can be created. These model-based protocols can be more effective than the current ones, since they provide individual treatment for the patients.

The article discusses two results. First is to find *an appropriate mathematical model for tumor volume evaluation from caliper-measured data*. Tumor as a 3-D object has three diameters (width, length and height); however in vivo only two diameters can be measured by caliper (width, *w* and length, *l*). To evaluate tumor volume, first the shape of the tumor has to be assumed, and if it is necessary for volume calculation, the third diameter (height, *h*) has to be approximated. According to the Xenograft tumor model protocol [20], tumor volume has to be calculated using the following formula:

$$\begin{array}{c} V=w^{2}\cdot \frac{l}{2}. \end{array}$$ (1)

The advantage of this model is that there is no need to approximate tumor height (which could result in error).

In several recent studies ([21, 22]), tumor volume is calculated assuming ellipsoid shape:

$$\begin{array}{c} V=\frac{4}{3}\cdot \pi \cdot \frac{l}{2}\cdot \frac{w}{2}\cdot \frac{h}{2}. \end{array}$$ (2)

Whilst studies have shown that tumors can be better estimated with ellipsoid shape than using Eq 1, calculating the volume of an ellipsoid requires the knowledge of the third parameter. A possible solution for height approximation [23] is:

$$\begin{array}{c} h=\frac{2}{3}\cdot l. \end{array}$$ (3)

Another relatively new but not widely used approach to estimate tumor volume is to assume hemi-ellipsoid shape [24]. In this case, tumor volume has to be calculated in the following way:

$$\begin{array}{c} V=\pi \cdot \frac{l}{2}\cdot \frac{w}{2}\cdot \frac{h}{2}. \end{array}$$ (4)

This estimation has the same disadvantage as ellipsoid estimation, i.e. tumor height has to be approximated.

From the abovementioned methods, the consequence is that the promising new direction in tumor volume evaluation is the dimension reduction, namely to find a statistical constant which can replace the need of measuring the tumor height. A two-dimensional mathematical model was created from the experimental results of BALB/c mice with KHJJ tumor line [25]:

$$\begin{array}{c} V=\frac{\pi }{6}\cdot f\cdot {\left(l\cdot w\right)}^{3/2}, \end{array}$$ (5)

where *f* is a constant which belongs to a certain tumor type. This formula was the starting point of our examination to find an appropriate mathematical model.

The second result which is discussed in the article is *comparison of the effectiveness of bevacizumab administration in the case of protocol-based and quasi-continuous therapies*. The effectiveness strongly depends on the administration, and a drug which is effective on a molecular level can be applied in a less effective way because of the incorrectly chosen administration. Our hypothesis is that the effectiveness of a lower dosage with a quasi-continuous therapy can be comparable with the protocol therapy.

## Materials and Methods

### Ethics statement

The study was carried out in strict accordance with the recommendations in the Guide for the Care and Use of Laboratory Animals of the National Institutes of Health. The protocol was approved by the Hungarian Animal Experimental Research Ethics Council (Állatkísérleti Tudományos Etikai Tanács, ATET), Permit Number: 22.1/1159/3/2010. All surgery and sacrifice were performed under sodium pentobarbital anaesthesia (Nembutal, 70 mg/kg), and all efforts were made to minimize suffering. Animals were carried out in the most humane and environmentally sensitive manner possible; in addition the 3Rs principle (replacement, refinement, reduction) was adequately implemented according to the Directive 2010/63/EU of the European Parliament. Mice were monitored daily. Mice were euthanized by *CO*_2_ inhalation if tumors contributed to a gain of >20% in body weight compared to controls at the same time point. Mice were humanely sacrificed using cervical dislocation.

### Overview of the phases

**Fig 1:**
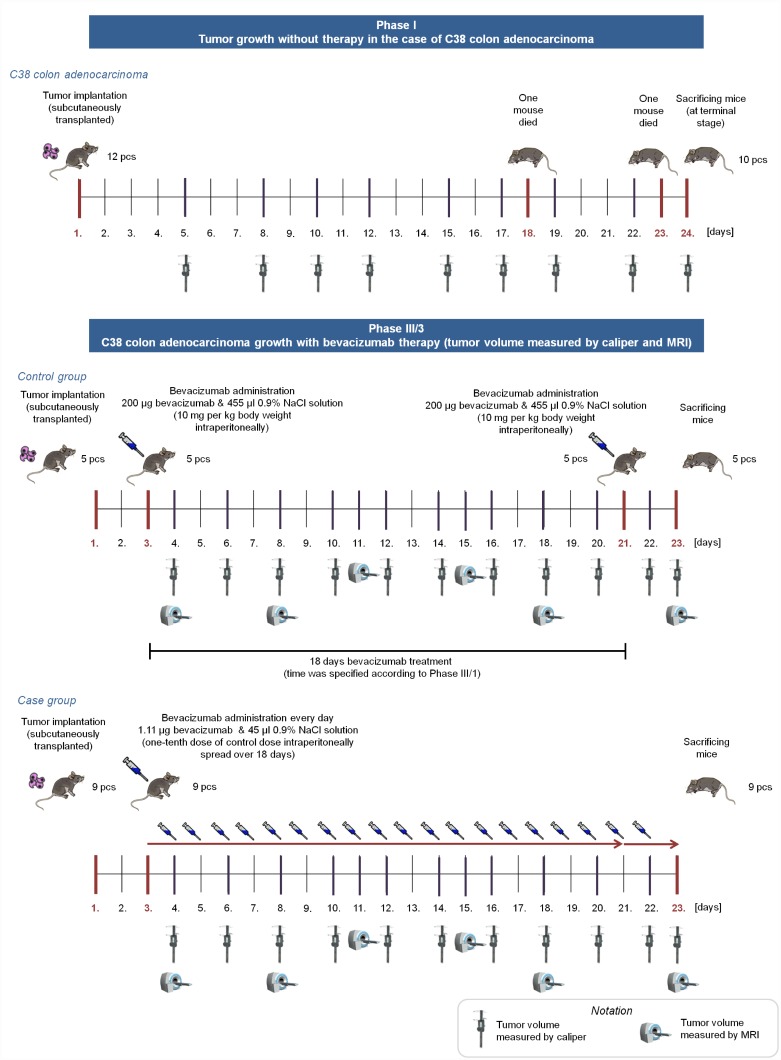
Overview of Phase I and Phase III/3. In Phase I, tumor growth was investigated without antiangiogenic therapy for 24 days (mice were sacrificed when the tumor reached a lethal size). In Phase III/3, control group members received one 200 *μg* bevacizumab dose for an 18-day therapy (on the 3rd and 21st days); case group members received 1.11 *μg* bevacizumab every day for 20 days.

The whole experiment consisted of four phases; the article focuses on the Phase III/3 and its results together with the comparison of Phase I and Phase III/3 (Fig 1).

In *Phase I*, we have investigated tumor growth without therapy with two types of mouse tumor. 12 immunocompetent mice were transplanted subcutaneously with C38 colon adenocarcinoma, and 11 immunocompetent mice were injected intramuscularly with B16 melanoma. Tumor volume was measured with digital caliper. Results of this phase can be found in [26].

In *Phase II*, the toxicology investigation of the applied angiogenic inhibitor (bevacizumab) was performed; there was no tumor implantation into mice in this phase. We monitored the vital parameters of 4 immunocompetent mice, and there was no serious toxic side-effect or lethality regarding to the usage of bevacizumab.

In *Phase III*, we have investigated C38 colon adenocarcinoma growth with bevacizumab therapy. Phase III contained three subphases, in every subphases two groups were created; control group received bevacizumab in one dose according to the protocol, while case group received substantially lower doses every day. In the first subphase (*Phase III/1*), control group members (5 immunocompetent mice) received 10 mg per kg body weight bevacizumab, while case group members (5 immunocompetent mice) received one-tenth dose of control dose spread over 18 days. Bevacizumab administration was started on the 7th day in both cases. Quantity of the optimal solvent administration was also examined in this subphase. Tumor volume was measured with digital caliper. The second subphase (*Phase III/2*) was similar to Phase III/1, mice received the same dosage of bevacizumab as in Phase III/1, however sample size was higher (6 immunocompetent mice in control group, 12 immunocompetent mice in case group), and bevacizumab administration started earlier, on the 3rd day of the experiment. Tumor volume was measured with digital caliper as well. Result of Phase III/2 was that the effectiveness of the small amount of quasi-continuous dosage is comparable with the effectiveness of one large dose [23], however this result is only based on caliper-measured data. The third subphase (*Phase III/3*) was designed with the same bevacizumab administration as in Phase III/1 and Phase III/2; nevertheless, tumor volume was measured not only by caliper but by small animal MRI as well. In this phase, 5 immunocompetent mice in control group, and 9 immunocompetent mice in case group were used.

**Fig 2:**
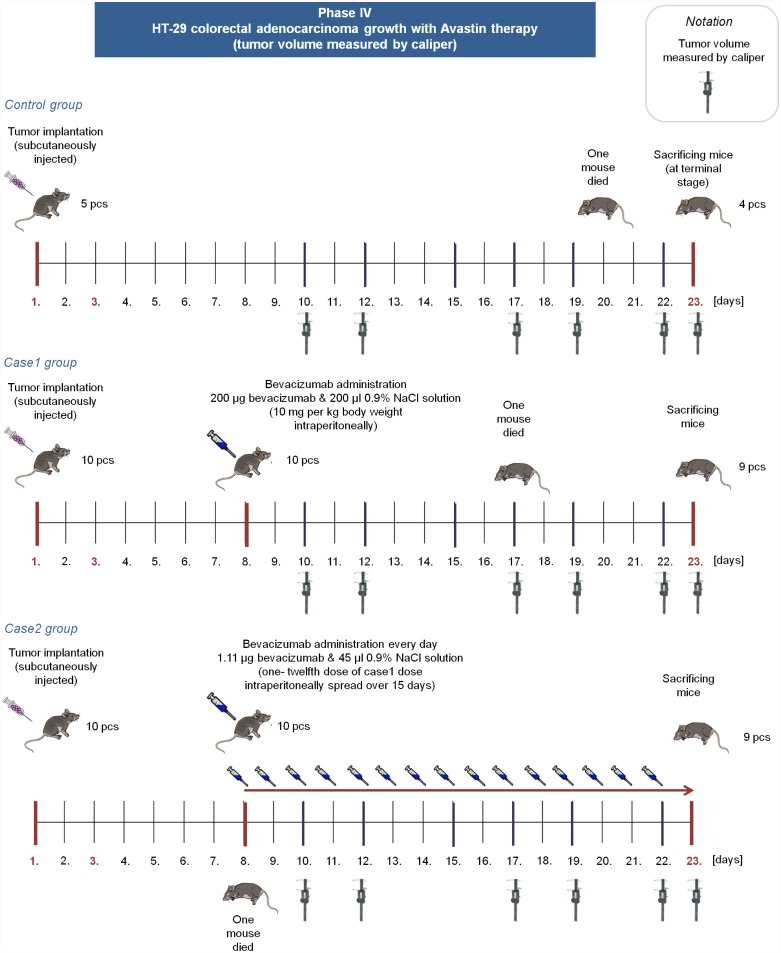
Overview of Phase IV. In Phase IV, control group members received no bevacizumab. Case1 group members received one 200 *μg* bevacizumab dose on the 8th day, case2 group members received 1.11 *μg* bevacizumab from the 8th day every day for 15 days.

Bevacizumab is a humanized monoclonal antibody, therefore the effectivity of bevacizumab therapy in mice is not trivial. Although there are studies which confirme that “bevacizumab is as effective as the murine anti-VEGF-R2 antibody (DC101) in mouse models” ([27, 28]), we extended our experiments and performed *Phase IV*, where immunocompromized (SCID) mice were used with human colorectal adenocarcinoma (HT-29) xenografts. In this phase, three groups were created. Mice in the control group received no treatment (5 mice); case1 group members (10 mice) received 10 mg per kg body weight bevacizumab in one dose; while case2 group members (10 mice) received 1/180 dose of control dose for 15 days. (Fig 2).

### Tumor implantation and bevacizumab administration

In Phase I, Phase II and Phase III, eight weeks old male C57Bl/6 mice with implanted C38 mouse colorectal carcinoma was used. In Phase IV, eleven weeks old male SCID mice with implanted HT-29 human colorectal adenocarcinoma was used. Mice were monitored daily by personnel experienced in recognizing signs of morbidity (illness, injury, or abnormal behavior) also in weekends and holidays. The C38 colorectal carcinoma line was maintained by serial subcutaneous transplantations in C57Bl/6 mice. (Inbred C57Bl/6 mice from our institute were used throughout the studies.) In Phase I, Phase II and Phase III, a piece of C38 colon adenocarcinoma was transplanted subcutaneously in the recipient animal on the 1st day of the experiment (approximately 2⋅10^6^ cells were transplanted into each mouse). In Phase IV, approximately 2.9⋅10^6^ cells were injected subcutaneously into each SCID mouse. Tumor was located in the last third of the back of the mouse in every case.

Mice in Phase I and Phase IV control group received no bevacizumab. Mice in Phase III/3 control and case groups, and mice in Phase IV case1 and case2 groups received bevacizumab. Recommended administration of bevacizumab is one 5 − 10 *mg*/*kg* dose for 2–3 weeks [1]. We have administered 10 *mg*/*kg* body weight intraperitoneally, which means 200 *μg* bevacizumab per a mouse, since the mass of the mice in the experiment was approximately 20 *g* (in the case of C57Bl/6 and SCID mice as well). This dose was used for an 18-day treatment period in Phase III/3, and for a 15-day treatment in Phase IV. In Phase III/3, control group members received 200 *μg* bevacizumab (with 455 *μl* 0.9% NaCl solution) in one dose intraperitoneally on the 3rd day and on the 21st day. In Phase III/3, case group members received one-tenth dose of control dose intraperitoneally spread over 18 days. It means that a mouse of the case group received 1.11 *μg* bevacizumab (with 45 *μl* 0.9% NaCl solution) every day from the 3rd day for 20 days. In Phase IV, case1 group members received 200 *μg* bevacizumab (with 200 *μl* 0.9% NaCl solution) in one dose intraperitoneally on the 8th day. In Phase IV, case2 group members received 1.11 *μg* bevacizumab (with 45 *μl* 0.9% NaCl solution) every day form the 8th day for 15 days. The reason for choosing this type of administration was as follows: a) we wanted to use a daily dose in order to investigate the biological effect of frequent impulse-like control signal for the future controller design; b) we wanted to examine such a low dose which has practically no side-effect but presumably is still effective.

### Tumor volume measurement

Tumor volume measurement cannot happen right after tumor implantation. In the case of C38 colon adenocarcinoma, first, the subcutaneously transplanted piece of tumor has to disintegrate, and after that the new tumor colony (which needs to be measured) can begin to grow from the disintegrated tumor cells. This process takes 4–5 days. The first measurement in Phase I occurred on the 5th day; in Phase III/3, it occurred on the 4th day. In Phase IV, the first measurement occurred when the tumors have reached an average volume of 50–60 *mm* ^3^ (according to the xenograft protocol [20]). It was on the 10th day, since HT-29 colorectal adenocarcinoma grows slower in mice than C38 colon adenocarcinoma.

Tumor volume was measured in two different ways. First way is the caliper measurement; in that case tumor diameters (width, length) are measured with digital caliper. It can be carried out in vivo during the experiment due to the subcutaneous localization of the tumor. Tumor volume (and the third diameter) has to be approximated, assuming a certain shape for the tumor. Measurements with caliper were done on the 5th, 8th, 10th, 12th, 15th, 17th, 19th, 22nd and 24th days of the experiment in Phase I; in the case of Phase III/3, they were done on the 4th, 6th, 8th, 10th, 12th, 14th, 16th, 18th, 20th, 22nd and 23rd days. In Phase IV, the caliper measurement days were the 10th, 12th, 17th, 19th, 22nd and 23rd days.

The other way to measure tumor volume is the usage of small animal MRI, a non-invasive in vivo technology giving the possibility of a more precise volume measurement [29]. In the experiment 9.4 Tesla field strength Varian small-animal MRI was used. Isofluorane (0.95 x 2.0%) was applied for inhalational anesthesia, and intubation was performed. Catheter was placed in the tail vein for injection—according to the mouse tail vein injection protocol [30]—to investigate drug effect. Position of the mouse was fixed to minimize the movement of the animal. Tumor was located in the last third of the back in every cases, thus the effect of respiratory movement was minimal. During the MRI measurements, life parameters of the mice were monitored. Breathing was monitored with piezoelectric transducer; temperature of the body was measured by rectal thermometer. The produced images were converted to NIfTI (Neuroimaging Informatics Technology Initiative) format, which is suitable for image processing. Tumor area was determined with flood fill algorithm [31] from the slides; by knowing the volume of a voxel, tumor volume was calculated from these two values.

**Fig 3:**
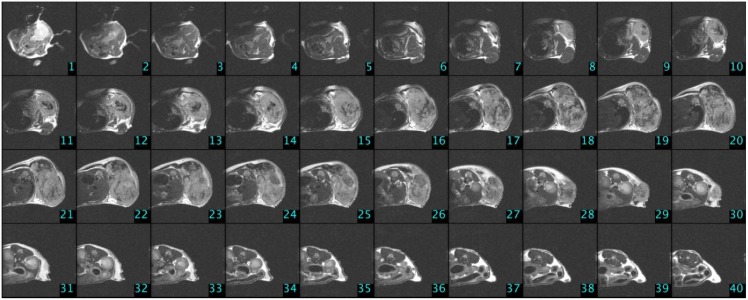
MRI slices in the case of a control group mouse (*C4*) on the 23rd day of the experiment (Phase III/3).

Contrast agents improve the visibility, but it is an extra strain to the organism, which can be lethal to animals which are in the final stage of cancer. We have found in our previous study [32] that measurements without contrast agents resulted in high quality images, where tumor can be circumscribed precisely, thus the usage of contrast is unnecessary. In the case of Phase I and Phase IV, there were no MRI measurements. Measurements with small animal MRI were done on the 4th, 8th, 11th, 15th, 18th and 23rd days of the experiment in Phase III/3. In the first four measurement times 30 slices were done from each mouse; in the last two measurement times 40 slices were done from each mouse due to the larger tumor volume. One can see experimental settings in Table 1, while MRI image of a control group mouse (*C4*) that was measured at the end of the experiment can be found in Fig 3.

**Table 1:** Experimental settings for small animal MRI measurement.

**30 slices**	**40 slices**
Pulse sequence: T1WI, sequence: FSE,	Pulse sequence: T1WI, sequence: FSE,
FOV: 25 x 25, spatial resolution: 128 x 128 x 30,	FOV: 30 x 30, spatial resolution: 128 x 128 x 40,
voxel size: 0.1953 x 0.1953 x 1 mm,	voxel size: 0.2343 x 0.2343 x 1 mm,
TR: 1800 ms, ESP: 7 ms,	TR: 2500 ms, ESP: 7 ms,
axial orientation: axial 90°, flip angle: 90°,	axial orientation: axial 90°, flip angle: 90°,
averages: 12, scan time: 5 min 53 s	averages: 10, scan time: 6 min 45 s

In the first four measurement times 30 slices, in the last two measurement times 40 slices were done from each mouse. Abbreviations: T1WI: T1 weighted image, FSE: Fast Spin Echo, FOV: Field of View [*mm* ^2^], TR: Repetition Time, ESP: Echo Spacing.

### Sacrificing mice, immunohistochemistry

Clinical aspects which have to be considered to determine when animals should be euthanized are body weight, physical appearance, measurable clinical signs, unprovoked behavior and response to external stimuli. Clinical criteria that establish when the endpoint have to be carried out: a) if tumors contributed to a gain of >20% in body weight compared to controls at the same time point; b) tumors that ulcerate, become necrotic or infected; c) any animal found unexpectedly to be moribund, cachectic, or unable to obtain food or water. All surgery and sacrifice were performed under sodium pentobarbital anaesthesia (Nembutal, 70 mg/kg). Mice were humanely sacrificed using cervical dislocation. No animals were sacrificed before the experimental endpoint. The size of maximum tumor volumes observed in our study fulfills the requirements of Hungarian animal experimental research protocol.

**Fig 4:**
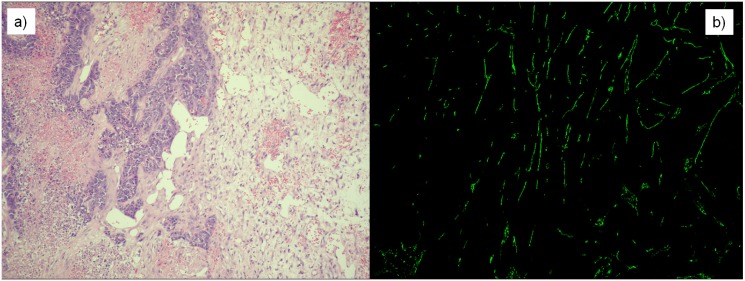
Stained slices in the case of *n1* mouse (Phase I, 24th day of the experiment). a) Haematoxylin Eosin (H&E) staining was applied to investigate tumor morphology. b) Fluorescence picture was created using CD31 antibody immunohistochemistry staining to calculate vascularization area.

In Phase I, mice were sacrificed when the tumor reached a lethal size; it was on the 24th day of the experiment. In the case of Phase III/3 and Phase IV, period of time was specified according to the previous phases (Phase I and Phase III/1); mice were sacrificed on the 23rd day of the experiment. After sacrificing mice, tumors were removed, and their mass was measured. Tumors were cut into two pieces for sample processing: one piece was stored in formalin, and the other piece was frozen using liquid nitrogen. Tumor morphology was investigated using standard Haematoxylin Eosin staining on the samples stored in formalin. Frozen samples were used to create 15 *μm* frozen cuts. After fixing in methanol, rat anti-mouse CD31 antibody (1:50, 550274 BD PHARMINGEN) was applied as primer antibody, and FITC conjugated anti-rat CD31 antibody (1:100, Jackson Immunoresearch, 712-095-150) was applied on the slides as secondary antibody. After staining, fluorescence pictures were done from the slides using confocal microscope (BIO-RAD MRC-1024). These images were applied to calculate vascularization area by using ImageJ (NIH, USA) [33] software (Fig 4).

### Statistical analysis

To compare the effectiveness of the different investigated treatment methods, statistical analysis was used. PASW Statistics 18 (SPSS Statistics, IBM, USA) and Matlab R2009b (MathWorks, USA) were used for statistical analysis. Normality was investigated with one-sample Kolmogorov-Smirnov test, and homogeneity of variance (homoscedasticity) was examined with Levene’s test. Analysis of Variance (ANOVA) test was used to compare more than two samples, while pairwise comparison was done by Tukey’s honest significant difference (HSD) test. Correlation was investigated with Pearson’s rank correlation test (Pearson’s *R*) and coefficient of determination (*R* ^2^).

## Results

In Phase I, 12 mice were implanted with C38 colon adenocarcinoma. One of them died on the 18th day, and another one on the 23rd day. The cause of death was tumor toxicity; however, none of the clinical criteria for euthanasia could be observed at these animals. In Phase I, 10 mice were sacrificed at the 24th day of the experiment. In Phase III/3, control group contained 5 mice, case group contained 9 mice; all mice were implanted with C38 mouse colon adenocarcinoma. No mice died during the experiment, therefore 5 mice from the control group, and 9 mice from the case group were sacrificed on the 23rd day. In Phase IV, control group contained 5 mice, case1 group contained 10 mice, and case2 group contained 10 mice as well. All mice were implanted with HT-29 human colorectal adenocarcinoma. In the control group, one mouse died on the 20th day due to tumor toxicity. In the case1 group, one mouse died on the 17th day. Autopsy revealed large metastasis in the lung, this is the suspected cause of the death. In the case2 group, one mouse died on the 8th day, before the therapy (bevacizumab administration) started. Autopsy revealed only minimal local tumor; however death was not caused by tumor.

### Comparing the results of caliper and MRI measurements; finding an appropriate mathematical model for tumor volume evaluation from caliper-measured data

In Phase III/3, MRI-measured tumor volume values are available which can be used as reference values for caliper-measured data. Applying the two-dimensional mathematical model (described in Eq 5) the goal is to find the *f* constant which belongs to the C38 colon adenocarcinoma and the treatment type. Starting with *f* = 1, we have investigated the goodness of the fitting, using an iterative method. Our results for the case group (daily, quasi-continuous small amount administration) and the control group (one big dose according to the protocol) are

$$\begin{array}{c} V_{pIII/3\_case}=\frac{\pi }{6}\cdot 6.08\cdot {\left(l\cdot w\right)}^{3/2}\to f_{pIII/3\_case}=6.08, \end{array}$$ (6)

$$\begin{array}{c} V_{pIII/3\_control}=\frac{\pi }{6}\cdot 3.68\cdot {\left(l\cdot w\right)}^{3/2}\to f_{pIII/3\_control}=3.68. \end{array}$$ (7)

**Fig 5:**
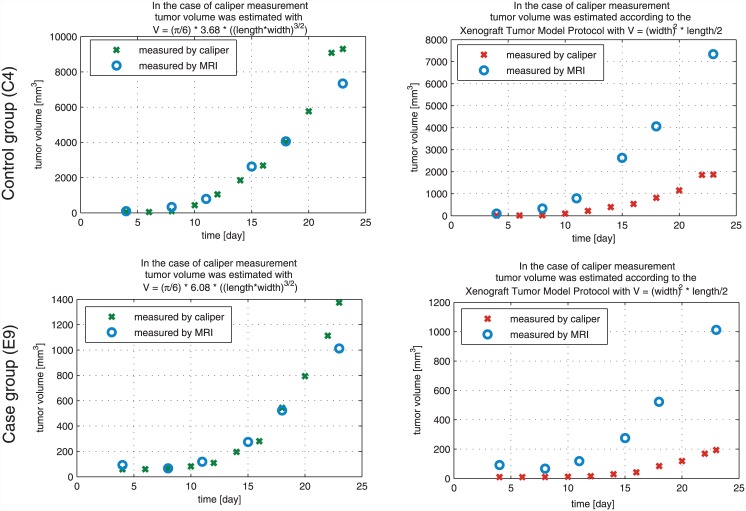
Validation of caliper-measured data. The figure shows the results of a mouse (*C4*) from control group (first row), and a mouse (*E9*) from case group (second row). The first column shows the tumor values which were calculated using the two-dimensional mathematical model; the second column represents the protocol-based tumor volumes. In each case the reference value is the MRI-measured tumor volume. One can see that the two-dimensional mathematical model fits to the MRI-measured values, while the protocol-based values present totally different curve.

Usage of this formula to calculate tumor volume from length and width values resulted in much more precise approximation of the MRI-measured tumor volume than protocol-based calculation. Numerical result can be found in Table 2, and Fig 5 presents graphical results.

**Table 2:** Experimental data of C38 mouse colon adenocarcinoma (tumor length, tumor width, tumor mass and tumor volume).

**Phase III/3 control group (23rd day)**						
Code of the mouse	Tumor length^a^ [*mm*]	Tumor width^a^ [*mm*]	Tumor mass^a^ [*g*]	Tumor volume caliper, protocol^b^ [*mm* ^3^]	Tumor volume caliper, 2-D model^b^ [*mm* ^3^]	Tumor volume MRI^c^ [*mm* ^3^]
C1	15.3	11.4	3.38	994	4439	3666
C2	26.5	18.4	8.67	4486	20746	9239
C3	13.0	9.4	2.11	574	2603	2081
C4	21.8	13.1	7.05	1871	9299	7335
C5	10.8	11.6	2.58	727	2702	2726
**Phase III/3 case group (23rd day)**						
Code of the mouse	Tumor length^a^ [*mm*]	Tumor width^a^ [*mm*]	Tumor mass^a^ [*g*]	Tumor volume caliper, protocol^b^ [*mm* ^3^]	Tumor volume caliper, 2-D model^b^ [*mm* ^3^]	Tumor volume MRI^c^ [*mm* ^3^]
E1	7.6	6.4	1.08	284	1080	1129
E2	9.1	6.6	0.98	390	1482	924
E3	10.7	10.0	2.34	927	3524	2707
E4	11.5	8.4	2.10	795	3023	2480
E5	10.3	8.4	1.95	674	2562	2226
E6	14.3	9.0	2.03	1223	4648	1929
E7	11.6	7.0	1.57	613	2329	1930
E8	19.7	14.3	5.00	3961	15052	5243
E9	8.4	6.8	0.86	362	1374	1013
**Phase I (24th day)**						
Code of the mouse	Tumor length^a^ [*mm*]	Tumor width^a^ [*mm*]	Tumor mass^a^ [*g*]	Tumor volume caliper, protocol^b^ [*mm* ^3^]	Tumor volume caliper, 2-D model^b^ [*mm* ^3^]	Tumor volume MRI^*d*^ [*mm* ^3^]
n1	21.9	15.1	7.22	2497	8029	7631
n2	13.8	10.2	2.81	718	2230	3011
n3	15.1	10.8	4.34	881	2781	4614
n4	23.0	15.1	8.05	2622	8642	8501
n5	25.4	15.8	10.43	3170	10734	10995
n6	19.1	13.8	5.57	1819	5714	5903
n7	exit: 18th day					
n8	exit: 23rd day					
n9	20.7	14.9	5.65	2298	7232	5987
n10	17.5	12.1	4.93	1281	4114	5232
n11	18.2	12.3	3.91	1377	4472	4164
n12	23.1	13.7	5.97	2168	7517	6322

Data was measured at the final day of Phase I (24th day) and Phase III/3 (23rd day).

^a^ Directly measured data (tumor length, tumor width, tumor mass)

^b^ Estimated data (tumor volume measured by caliper, calculated according to Xenograft tumor model protocol or two-dimensional mathematical model)

^c^ MRI-measured data (tumor volume calculated with flood fill algorithm)

^d^ Evaluated data (“MRI” tumor volume calculated from linear curve fit (see Eq 8))

**Fig 6:**
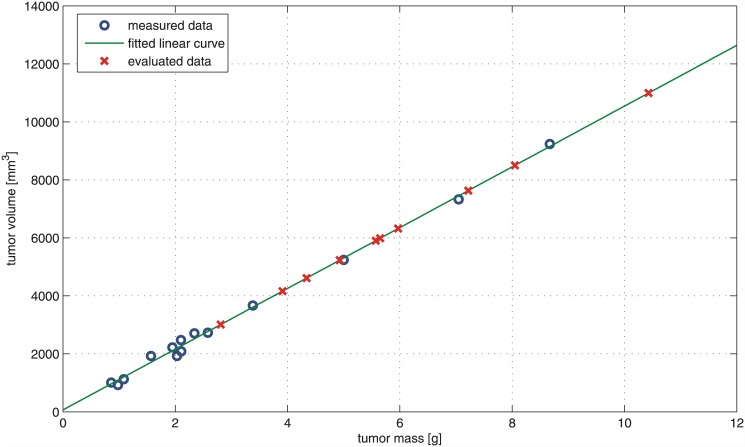
Evaluation of Phase I tumor volume values. “Measured data” is the MRI-measured tumor volume—tumor mass pairs on the 23rd day of Phase III/3 (case and control group). For this dataset, linear curve fitting was carried out (“fitted linear curve”) to find the mathematical relationship between MRI-measured tumor volume and tumor mass. Substituting tumor mass values which were measured on the 24th day of Phase I to the equation of the resulted curve, the corresponding tumor volume values can be evaluated (“evaluated data”).

To find the *f* constant for tumor growth without therapy (Phase I), first reliable tumor volume values had to be found, since there was no MRI measurement in Phase I. Beside tumor diameters, tumor mass was measured and vascularization area was calculated in the case of the removed tumors. We have investigated the relationship between MRI-measured tumor volume and vascularization area (Phase III/3 case and control groups, 23rd (final) day of the experiment) but no significant correlation was found (same results were published in the case of Phase I [26] and Phase III/2 [23]). Examining the relationship between MRI-measured tumor volume and tumor mass values, we have found a very strong linear correlation (*R* = 0.998, *R* ^2^ = 0.996, *p* < 0.0001). It means that knowing the tumor mass, tumor volume can be estimated with suitable accuracy; hence the lack of MRI measurement can be replaced in the case of Phase I. In the light of the above mentioned, linear curve fitting was carried out to find the mathematical relationship between MRI-measured tumor volume and tumor mass (Phase III/3 case and control groups). The resulted linear curve is

$$\begin{array}{c} v=1047.7m+67.1, \end{array}$$ (8)

where *v* is tumor volume [*mm* ^3^] and *m* is tumor mass [*g*]. Substituting tumor mass values which were measured in Phase I into Eq 8, the corresponding tumor volume values can be evaluated (one can find numerical results in Table 2 *Tumor volume “MRI”* column; and graphical results in Fig 6). The last step is to find the *f* constant of the two-dimensional mathematical model for tumor growth without therapy (Phase I). Using the above mentioned iterative method, the resulted equation is

$$\begin{array}{c} V_{pI}=\frac{\pi }{6}\cdot 2.55\cdot {\left(l\cdot w\right)}^{3/2}\to f_{pI}=2.55. \end{array}$$ (9)

**Fig 7:**
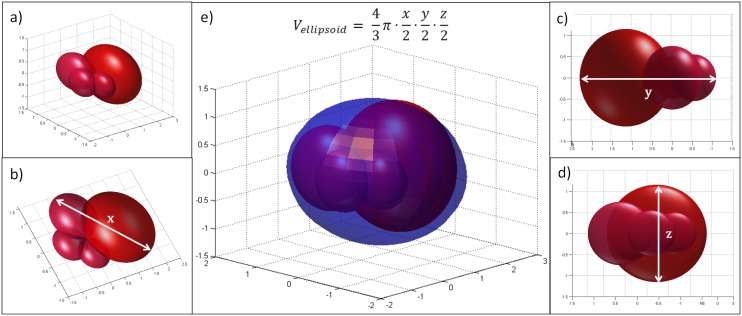
Illustration for tumor with irregular structure (berry-shaped). a) berry-shaped tumor; b) *x*-diameter of the tumor; c) *y*-diameter of the tumor; d) *z*-diameter of the tumor; e) berry-shaped tumor with ellipsoidal estimation. Even though all the three diameters can be measured, the estimation of the volume has quite a large error.

One can see from Table 2 that the goodness of the fit is different in the case of Phase I (tumor growth without therapy) and in the case of Phase III/3 (tumor growth with antiangiogenic therapy). Investigating the results of Phase III/3 one can observe that the two-dimensional mathematical model has good estimation property when the tumor width and length values are small; however, for large tumor diameter values the estimation could result in significant error, the estimated value is greater than the measured one (outliers are *E8*, *C2*). In the case of Phase I, no similar problem occurs; the two-dimensional mathematical model can handle great values as well (e.g. *n5*). This problem can be explained by our observation, namely tumors which were grown without therapy have more symmetric and solid closed shape, in contrast to tumors which were grown under antiangiogenic therapy. We have found that mice which have received therapy had tumor with irregular, and in several cases berry-shaped structure, especially when reaching large volume. In that case—even though all the three diameters can be measured—the estimation of the volume has quite a large error. A 3-D illustration can be found in Fig 7.

### Comparing the effectiveness of bevacizumab administration in the case of protocol-based and quasi-continuous therapies

The results of the three investigated cases using C38 mouse colon adenocarcinoma (Phase I, Phase III/3 control group, and Phase III/3 case group) were compared using tumor volume values from MRI measurements (Phase III/3 control and case groups, 23rd day) and evaluated data (Phase I “MRI” tumor volume calculated from linear curve fit, 24th day). One can find datasets in the last column of Table 2. Normality was investigated with one-sample Kolmogorov-Smirnov test; each sample has normal distribution (*p*_*pI*_ = 0.883, *p*_*pIII*/3_*case*_ = 0.716, *p*_*pIII*/3_*control*_ = 0.869). Homogeneity of variance was examined with Levene’s test; the sample variances are equal (*p* = 0.052).

**Fig 8:**
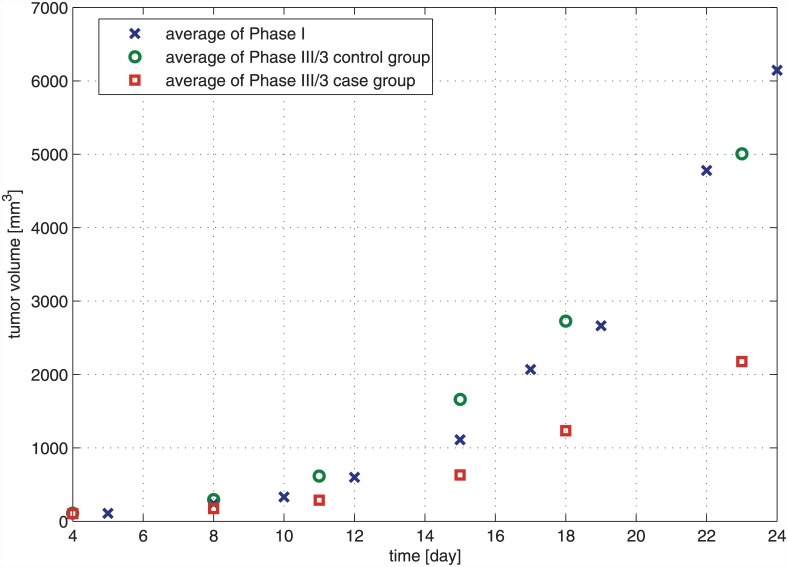
Average of tumor volumes for every measurement days of the experiment in the case of Phase I, Phase III/3 control and Phase III/3 case group. The significant difference between quasi-continuous therapy (Phase III/3 case group) and tumor growth without treatment (Phase I) was proved with statistical analysis as well.

To compare more than two samples, ANOVA test was applied. We have found that there is significant difference between the means of the samples (*p* = 0.002, using 0.05 level of significance). Pairwise comparison was done by Tukey’s honest significant difference test to find those samples, which have significantly different means. The post hoc test resulted in the following. Phase I and Phase III/3 control groups are not significantly different (*p* = 0.572), while Phase I and Phase III/3 control groups are significantly different (*p* = 0.002). This means that mice which were treated with the recommended bevacizumab protocol (one 200 *μg* bevacizumab dose for an 18-day therapy) did not have significantly smaller tumor volume than mice which did not receive therapy at all. However mice which were treated with a quasi-continuous therapy (one-tenth dose of control dose spread over 18 days, i.e. 1.11 *μg* bevacizumab every day) had significantly smaller tumor volume than mice that did not receive therapy. Average of tumor volumes for every measurement days of the experiment can be seen in Fig 8.

To validate our results which come from the experiments where mouse tumor vascularization was inhibited with humanized VEGF, we evaluated the results of Phase IV, where immunocompromized (SCID) mice were used with human colorectal adenocarcinoma (HT-29) xenografts. In this phase, control group members received no treatment; case1 group members received one 200 *μg* bevacizumab dose; while case2 group members received 1.11 *μg* bevacizumab daily for 15 days (the total dose of case2 group members is the one-twelfth dose of case1 group members). For the most precise result, tumor mass values, which were measured at the end of the experiment, were compared in the statistical analysis. One can find datasets in Table 3. Pursuant to one-sample Kolmogorov-Smirnov test, each sample has normal distribution (*p*_*pIV*_*control*_ = 0.981, *p*_*pIV*_*case*1_ = 0.998, *p*_*pIV*_*case*2_ = 0.854). Levene’s test showed that the sample variances are equal (*p* = 0.108). The means of the samples are *m*_*pIV*_*control*_ = 0.65, *m*_*pIV*_*case*1_ = 0.70, *m*_*pIV*_*case*2_ = 0.46. ANOVA test showed significant difference between these means (*p* = 0.041, using 0.05 level of significance). Tukey’s HSD test resuled in significant difference between case1 and case2 groups (*p* = 0.038). This means that the daily treatment with one-twelfth total dose resulted in significantly smaller tumors than the protocol-based treatment.

**Table 3:** Experimental data of human colorectal adenocarcinoma (HT-29) xenografts (tumor mass).

**Phase IV control group (23rd day) tumor mass [g]**	**Phase IV case1 group (23rd day) tumor mass [g]**	**Phase IV case2 group (23rd day) tumor mass [g]**
0.73	0.96	0.64
0.66	0.54	0.60
0.39	0.72	0.42
0.81	0.80	0.37
	1.13	0.43
	0.40	0.45
	0.80	0.41
	0.61	0.53
	0.37	0.29

## Discussion

Since 2004, the target therapy of bevacizumab (Avastin) is widely used to treat colorectal [9], kidney [10], cervical [34], ovarian [35], non-small cell lung cancers [5], melanoma [14] and certain brain tumors (e.g. recurrent glioblastoma (rGBM) [36]) as a first or second line treatment, usually in combination with chemo- or immunotherapy. The usual administration is via intravenous infusion; once every 2 or 3 weeks. The dose depends mainly on weight. However, most serious questions are: for how long and continuous or not? The most recent ESMO (European Society for Medical Oncology) consensus guidelines suggest that treatment discontinuation or maintenance are feasible options after 4–6 months of full-dose first-line therapy to treat colon and rectal adenocarcinoma [37]. However, if the treatment is lengthy, the problem of side-effects also has to be considered. To overcome all of these difficulties (continuity of the administration and side-effects), model identification should be determined and then a control algorithm (controller) can be designed for the created mathematical model. Of course, for the closed-loop design frequent and precise tumor volume measuremets are required, thus the problem of tumor volume measurement has to be solved as well. Finding the mathematical relationship between MRI-measured tumor volume and tumor mass creates the possibility to estimate tumor volume from caliper-measured data. However, it has to be taken into consideration that in several cases using antiangiogenic therapy, tumor shape is irregular (berry-shaped). Consequently, when tumor mass data is unavailable (during the experiment), tumor volume value can be validated with MRI; in that way outlier data points (which were calculated using the two-dimensional mathematical model) can be filtered out. In clinical practice, the determination of the tumor size (volume) is done by MRI and/or CT, but this is for the purpose of validating the effectiveness of the treatment, and therefore it is not a continuous monitoring. In the concept of model-based treatment, a continuous and precise tumor volume monitoring is needed concerning which, may be possible in the near future by nanotechnology [38].

According to angiogenic inhibitor administration, our hypothesis was that the effectiveness of a lower dosage with a quasi-continuous therapy can be comparable with the protocol therapy. Nevertheless the result of our study shows that the effectiveness is even better. Consequences are manifold. First of all, our hypothesis according to the importance of administration is proved. The effectiveness of the quasi-continuous (daily) 1/180 dosage (1.11 *μg* relative to 200 *μg*) using C38 mouse colon adenocarcinoma was more effective than one large dose. We found the same result for human colorectal adenocarcinoma (HT-29) xenografts. The explanation of the effectiveness of low dose VEGF therapy is the following ([39–44]). Healthy tissues have efficient vasculature and effective blood supply due to the balance of proangiogenic and antiangiogenic factors. When tumor cells become able to produce proangiogenic factors (the process is called as angiogenic switch), it will result in abnormal and inefficient vascular network (with high vascular permeability and poor perfusion) due to the hurried process of vessel formation. Tumor growth can take place under such conditions; however delivery of chemotherapeutic drugs is obstructed. Hence, the first step has to be the vasculature normalization with the reconstruction of the balance of pro- and antiangiogenic factors. This means that continuous, low dose antiangiogenic factor (VEGF) administration is needed. This creates the possibility of further efficient therapeutic agent use (if any) or assists the more effective direct antitumoral effect of bevacizumab.

Similarly, Zhang et al. [45] have found that the combination of low-dose cyclophosphamide and ginsenoside Rg3 therapy can be more effective than the normal administration. Although the low dose bevacizumab therapy is well known in the literature, we found no article (in the English literature) regarding very low dose and quasi-continuous bevacizumab therapy, as used in our study. Secondly, the aspect of side-effects are not inconsiderable. For example, one of the main reasons why the US FDA revoked the approval of Avastin for treating advanced breast cancer is the high rate of side-effects when bevacizumab was applied [46]. Typical side-effects and adverse events are arterial and venous thromboembolic events, bleeding, hypertension, febrile neutropenia, infections, proteinuria, mucositis, and hand-foot syndrome. Using an extremely low dosage (as in our study), there is a high probability that these adverse effects can be minimized.

In our study, we applied a two-dimensional mathematical model and determined the corresponding tumor- and therapy-specific constants in the three investigated cases (C38 colon adenocarcinoma growth without therapy, C38 colon adenocarcinoma growth with protocol-based bevacizumab therapy and C38 colon adenocarcinoma growth with quasi-continuous bevacizumab therapy). This helps to calculate accurate tumor volume from caliper measured data which will be useful for further animal experiments and as a consequence no small MRI measurements will be needed. In addition, we have found that the quasi-continuous administration of bevacizumab is effective against tumor growth of C38 colon adenocarcinoma, in contrast to protocol-based treatment. Considering the possibility of precise tumor volume determination and the effective quasi-continuous drug administration, it opens a new treatment choice based on closed-loop control. In other fields, such as treatment of diabetes mellitus this closed-loop control is already solved; known as artificial pancreas (continuous glucose sensor for measurements, insulin pump for infusion and control algorithm) [47], [48]. Similarly, we may have the possibility to a) measure the tumor volume using nanotechnology, b) create a pump for drug administration and c) design control algorithms for specific tumor types. Controller-based therapy has the obvious advantage against e.g. constant low-dose therapy because in the case of controller-based administration, the control signal (administration dose) can vary from zero to the maximum tolerable dose according to the perceived tumor volume. In this way, the targeted therapy can be used in a much more individualized form.
